# Vaginal Lactobacilli Supernatants Protect from Herpes Simplex Virus Type 1 Infection in Cell Culture Models

**DOI:** 10.3390/ijms25052492

**Published:** 2024-02-20

**Authors:** Elisa Avitabile, Laura Menotti, Barbara Giordani, Vanessa Croatti, Carola Parolin, Beatrice Vitali

**Affiliations:** Department of Pharmacy and Biotechnology, University of Bologna, 40127 Bologna, Italy; elisa.avitabile@unibo.it (E.A.); barbara.giordani4@unibo.it (B.G.); vanessa.croatti2@unibo.it (V.C.); carola.parolin@unibo.it (C.P.); b.vitali@unibo.it (B.V.)

**Keywords:** vaginal lactobacilli, cultural supernatants, herpes simplex virus type 1, Vero cells, HeLa cells, virus growth inhibition

## Abstract

A healthy vaginal microbiota hosts *Lactobacillus* as the most predominant genus. Lactobacilli play a role in human health through the production of diverse antimicrobial substances that can act against human pathogens or modulate the immune system. Previous reports highlighted the ability of vaginal lactobacilli to counteract viruses causing STIs, e.g., HIV-1 and HSV-2. In this report, we analyze the activity of supernatants of vaginal lactobacilli against HSV-1 infection, which is becoming increasingly relevant as a STI. We show that the supernatants of two vaginal *Lactobacillus* species (i.e., *L. crispatus* and *L. gasseri*) were active at neutralizing HSV-1 infection in two different cell lines of human and simian origin. Specifically, we demonstrate that *L. crispatus* strains are the most effective in antiviral activity, as evidenced by the comparison with a vaginal pathogen taken as reference. The effect was specific and not attributable to the generic toxicity of the supernatants to the cells. Our results pave the way for the development of probiotics to limit the impact of HSV-1 infection on women’s health.

## 1. Introduction

The human body is inhabited by various microorganisms that establish a mutualistic relationship with the host; the different physiological characteristics of human niches shape the composition of specific human microbiotas that ultimately provide antipathogenic and functional activities to the human host [1]. The vaginal microbiota of healthy premenopausal women is characterized by low microbial diversity and is dominated by the *Lactobacillus* genus, with *Lactobacillus crispatus* and *Lactobacillus gasseri* being among the most frequent species [2,3,4]. Vaginal lactobacilli contribute to human well-being and reproductive health by impeding pathogen invasion through the production of different antimicrobial molecules, competitive exclusion, and modulation of the host immune system [5,6,7,8,9]. Active substances released by lactobacilli, like lactic acid, hydrogen peroxide, bacteriocins, and exopolysaccharides, proved to be effective against bacteria, fungi, and viruses [5,6,7]. Vaginal *Lactobacillus* strains isolated from healthy women demonstrated activity against viruses responsible for sexually transmitted infections (STIs), including human immunodeficiency virus type 1 (HIV-1) and herpes simplex virus type 2 (HSV-2) [10,11,12,13,14,15]. Due to these activities, the use of lactobacilli and/or their metabolites could represent an attractive strategy to prevent or treat different viral infections, especially STIs [16].

Nowadays, as the most recent comprehensive reports show, there is an increasing incidence of genital infections caused by herpes simplex virus type 1 (HSV-1) derived from contact with the mucosa from other body areas [17,18,19]. As an example, while there is a decreasing trend in the overall prevalence of HSV-1, in young adults, genital versus facial HSV-1 infection rates show an increasing trend and account for >50% of all new genital HSV infections [20]. HSV-1 is an enveloped, double-stranded DNA and neurotropic virus affecting 70% of the population worldwide [21,22]. HSV-1 generally infects different areas of the human body, such as the eyes, skin, mouth, throat, and central nervous system, and can cause diseases such as keratoconjunctivitis, herpetic whitlow, gingivostomatitis, pharyngitis, encephalitis, and meningitis [23,24,25]. It is estimated that 90% of the population is seropositive for this pathogen, and its transmission, which usually occurs in the early stages of life, requires direct contact with an HSV-1-positive individual [21,26].

After the primary infection at the level of epithelial cells, the virus reaches the nerve ganglia through the peripheral sensory nerves, establishing a latent infection. Periodically, when the immune system is challenged by stress factors, i.e., fever, ultraviolet light exposure, emotional stress, and the common cold, the virus can reactivate and cause recurrent HSV-1 disease [22]. Antivirals based on nucleoside analogues, such as acyclovir, valacyclovir, penciclovir, and famciclovir, are nowadays used as a first-line treatment for HSV-1 lesions. The major risk of using such drugs is the development of HSV-1-resistant mutants, which are especially frequent in immunocompromised patients [26,27]. Other drugs used as second-line antivirals, such as cidofovir and foscarnet, showed various toxic side effects [28]. The absence of a prophylactic strategy and the limited effectiveness of the current antiviral therapies point out the need for novel and safer anti-HSV-1 agents.

In previous research, the culture supernatants of vaginally isolated lactobacilli have been demonstrated to significantly suppress HIV-1 infection in human ex vivo tissues, with a virucidal effect [13]. Here, we investigated for the first time the anti-HSV-1 activity of culture supernatants collected from representative strains of the aforementioned vaginal lactobacilli collection, namely *Lactobacillus crispatus* BC3 and BC5, and *Lactobacillus gasseri* BC12 and BC13.

## 2. Results

### 2.1. Lactobacilli Supernatants Reduce HSV-1 Replication

**Figure 1:**
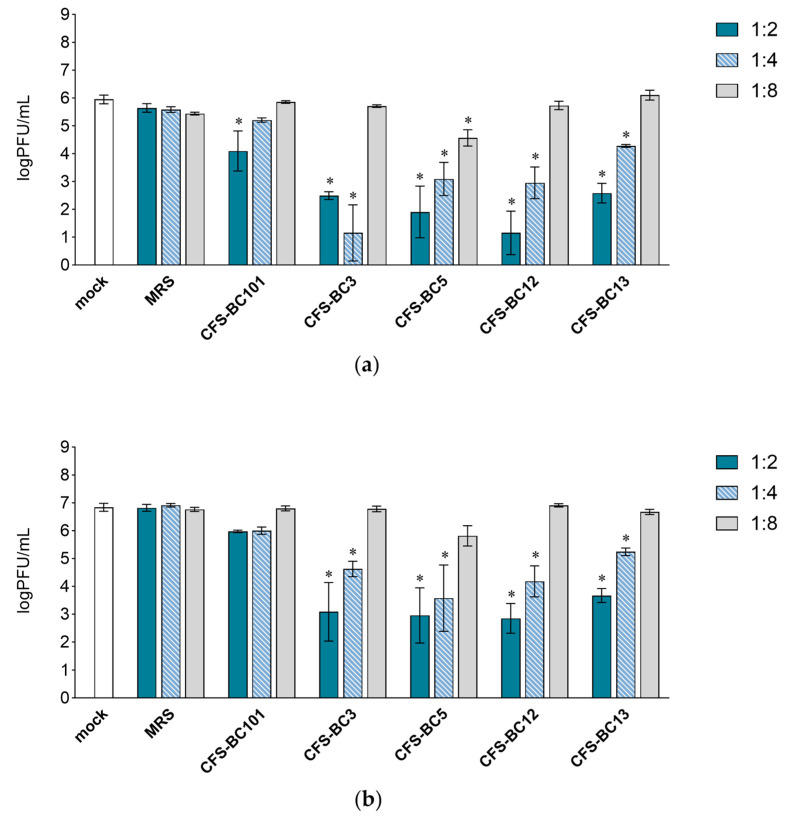
Antiviral activity of CFSs from *L. crispatus* BC3 and BC5, *L. gasseri* BC12 and BC13, *E. faecalis* BC101, and MRS medium on HeLa cells. HSV-1 was mock pretreated or pretreated with CFSs or MRS medium at different dilutions (1:2, 1:4, and 1:8), and virus yield was determined at (**a**) 24 h and (**b**) 48 h post-infection. Results are expressed as log PFU/mL (mean ± SD, *n* = 3). * *p* < 0.05 (vs. mock).

**Figure 2:**
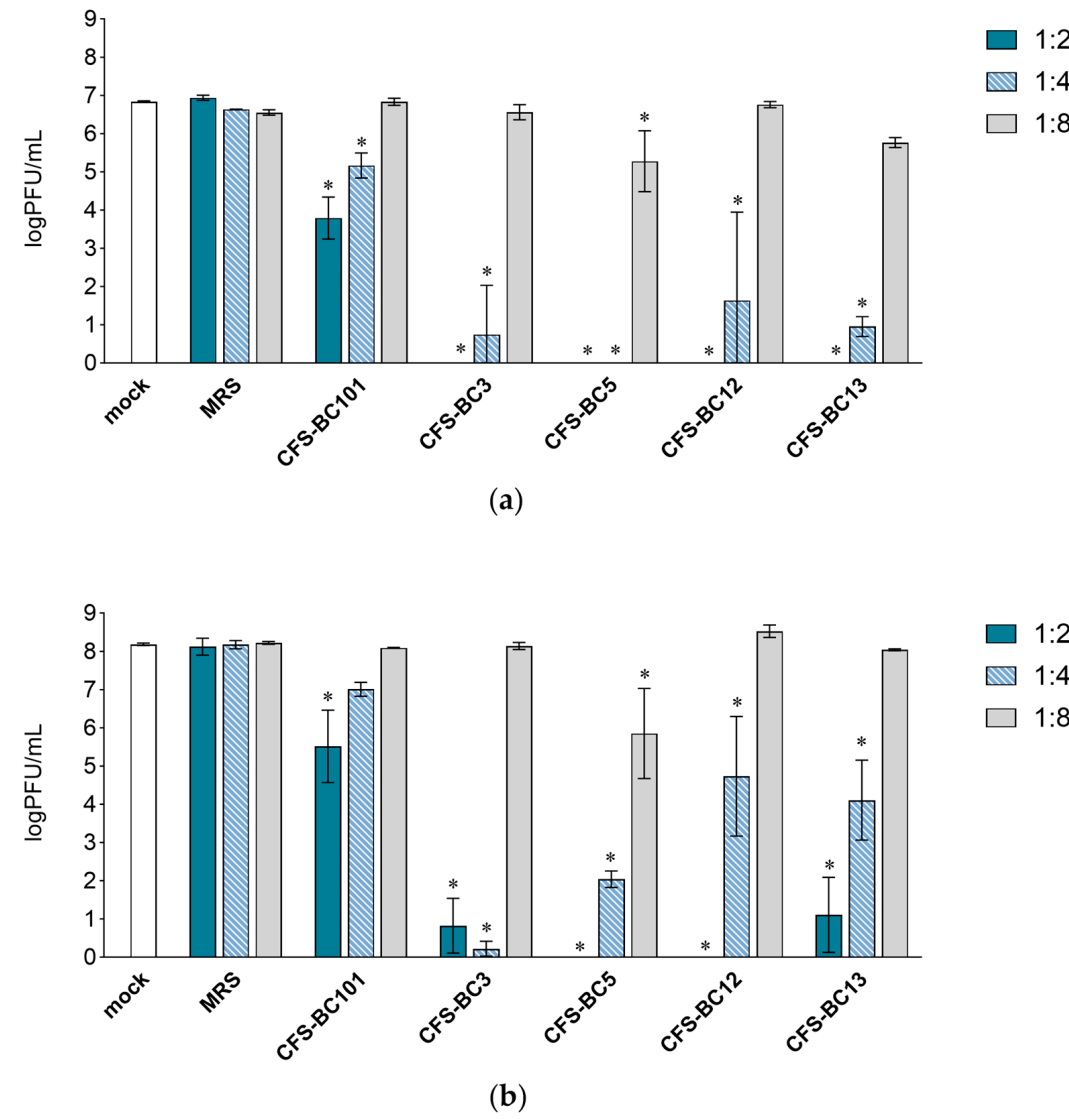
Antiviral activity of CFSs from *L. crispatus* BC3 and BC5, *L. gasseri* BC12 and BC13, *E. faecalis* BC101, and MRS medium on Vero cells. HSV-1 was mock pretreated or pretreated with CFSs or MRS medium at different dilutions (1:2, 1:4, and 1:8), and virus yield was determined at (**a**) 24 h and (**b**) 48 h post-infection. Results are expressed as log PFU/mL (mean ± SD, *n* = 3). * *p* < 0.05 (vs. mock).

To assess the antiviral effect of *Lactobacillus* cell-free supernatants (CFSs), two mammalian cell lines were used: Vero cells, one of the most used cell lines for HSV-1 manipulation, and HeLa cells, as human cells of the female genital tract, to recapitulate the in vivo setting. For virus inhibition assays, the conditions were standardized in terms of the number of cells and the multiplicity of infection to be able to compare different experiments. Time points were chosen according to the standard conditions for HSV-1 viral yield experiments: 24 h as the first point and 48 h when yield plateaus. Experiments were set as reported: to seek a possible dose-response correlation, two-fold serial dilutions of bacterial CFSs from vaginal *Lactobacillus* strains, namely *L. crispatus* BC3 and BC5, and *L. gasseri* BC12 and BC13, were prepared. Then, HSV-1 was added to each dilution for the pre-incubation treatment. To test the effect of bacterial culture medium or of generic bacterial products in supernatants, two control conditions were included: MRS medium not inoculated and a reference supernatant taken from cultures of an unrelated bacterial species, i.e., *Enterococcus faecalis* BC101. Cell cultures were monitored by light microscope observation before and after the end of adsorption. Experiments carried out in HeLa cells (Figure 1) showed a pattern of HSV-1 replication inhibition exerted by all CFSs, not by the MRS control. The inhibition mediated by CFSs showed a dose response effect, which could be observed already at 24 h post-infection (Figure 1a) but was fully visible at 48 h post-infection (Figure 1b). CFS from *L. crispatus* BC5 was the most effective, as it showed inhibition activity even at 1:8 dilution, whereas the other CFSs at 1:8 apparently were not effective, and virus yields reached the level of HSV-1 untreated control. The virus inhibition effect was even more evident in experiments carried out in Vero cells (Figure 2). In this setting, already at 24 h post-infection, *Lactobacillus* CFS samples (1:2 and 1:4 dilutions), especially CFS from *L. crispatus* BC5, showed a complete inhibition of viral replication (Figure 2a). *L. crispatus* BC5 CFS antiviral activity was maintained at 48 h post-infection, too, since the 1:8 dilution determined the lowest viral yield compared to all the other samples (Figure 2b). CFSs recovered from *L. crispatus* BC3 and *L. gasseri* BC12 displayed an antiviral effect that could be read up to the 1:4 dilution. In order to highlight the peculiar antiviral potential of lactobacilli in terms of the magnitude of the effect, we compared the inhibitory activity exerted by lactobacilli supernatants with that of *E. faecalis* BC101. In this analysis (lactobacilli versus *E. faecalis*), we considered only the experimental conditions for which lactobacilli CFSs had a significant inhibitory effect versus mock. The inhibitory activities exerted by CFSs from lactobacilli at dilutions 1:2 and 1:4 were always significantly higher than those observed for CFS from *E. faecalis* BC101 in the Vero cells model, both at 24 h and 48 h post-infection. CFSs from lactobacilli were more effective in inhibiting the infectivity of HSV-1 in HeLa cells, with a few exceptions (CFS-BC13 diluted 1:4 at 24 h and 48 h post-infection). CFS from *L*. *crispatus* BC5 determined lower viral yields in Vero and HeLa cells than CFS from *E. faecalis* BC101, even at dilution 1:8, confirming its antiviral potential. These data indicate that the antiviral activity of lactobacilli is significantly higher than that of the reference strain. No dilution effect was observed for MRS medium, neither in HeLa nor in Vero cells. The pretreatment of the virus with CFSs was performed identically on the two cell lines, but differences were observed in terms of the virus inhibition readout. The absolute viral yields in HeLa cells were about one order of magnitude lower than in Vero cells. This implies that the experiment in Vero cells was more sensitive, as Vero cells can amplify the incoming virus to higher titers.

### 2.2. Lactobacilli Supernatants Do Not Influence the Viability of Mammalian Cells

The MTT assay and erythrosine B exclusion test were performed in order to exclude any possible effect of CFSs from *L. crispatus* (BC3 and BC5), *L. gasseri* (BC12 and BC13), and *E. faecalis* (BC101) on the viability of Vero and HeLa cell lines. CFSs were diluted to reproduce the highest concentrations applied to cells in antiviral experiments, corresponding to an initial dilution of 1:2.

**Figure 3:**
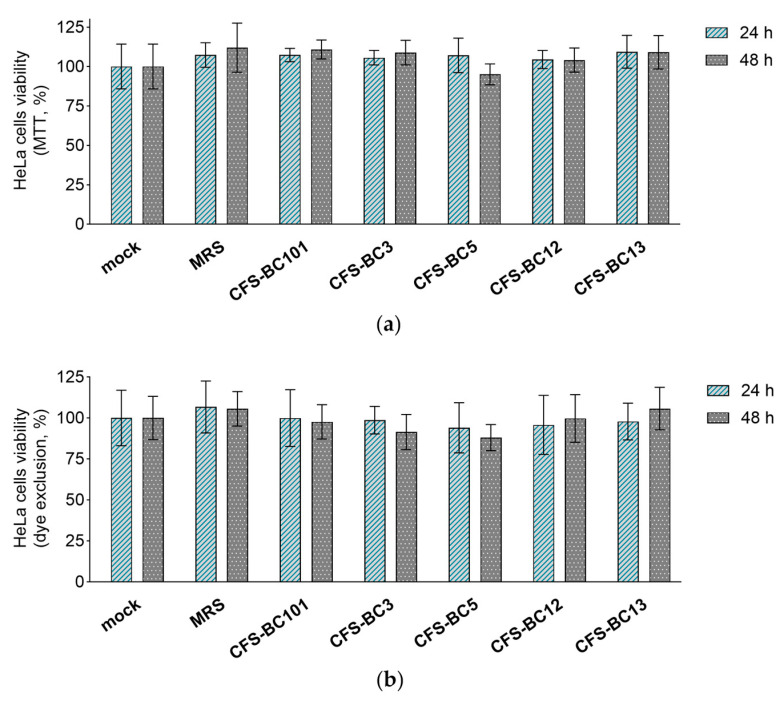
Effect of CFSs on HeLa cell viability at 24 h and 48 h after exposure. CFSs from *L. crispatus* BC3 and BC5, *L. gasseri* BC12 and BC13, *E. faecalis* BC101, and MRS medium were tested at the highest concentration used in the antiviral assay (corresponding to a 1:2 dilution) by means of (**a**) the MTT assay and (**b**) the erythrosine B exclusion test. Results are expressed as % of mock (100%) (mean ± SD, *n* = 3).

**Figure 4:**
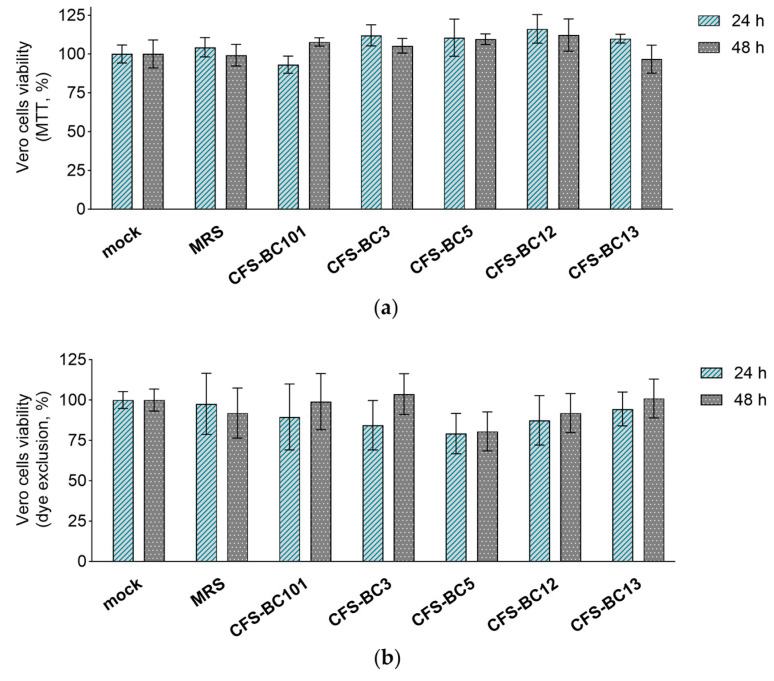
Effect of CFSs on Vero cell viability at 24 h and 48 h after exposure. CFSs from *L. crispatus* BC3 and BC5, *L. gasseri* BC12 and BC13, *E. faecalis* BC101, and MRS medium were tested at the highest concentration used in the antiviral assay (corresponding to a 1:2 dilution) by means of (**a**) the MTT assay and (**b**) the erythrosine B exclusion test. Results are expressed as % of mock (100%) (mean ± SD, *n* = 3).

Importantly, bacterial CFSs did not affect the mitochondrial activity (MTT assay) or cell proliferation (dye exclusion assay) after 24 h and 48 h exposure of HeLa (Figure 3) and Vero (Figure 4) cells, suggesting that they do not interfere with cell viability. These results underline that the antiviral activity observed in the presence of *Lactobacillus* CFSs was related to their ability to reduce HSV-1 infection and not to any effect on HeLa and Vero cell viability.

### 2.3. Overview of CFS-Mediated Antiviral Activity

**Figure 5:**
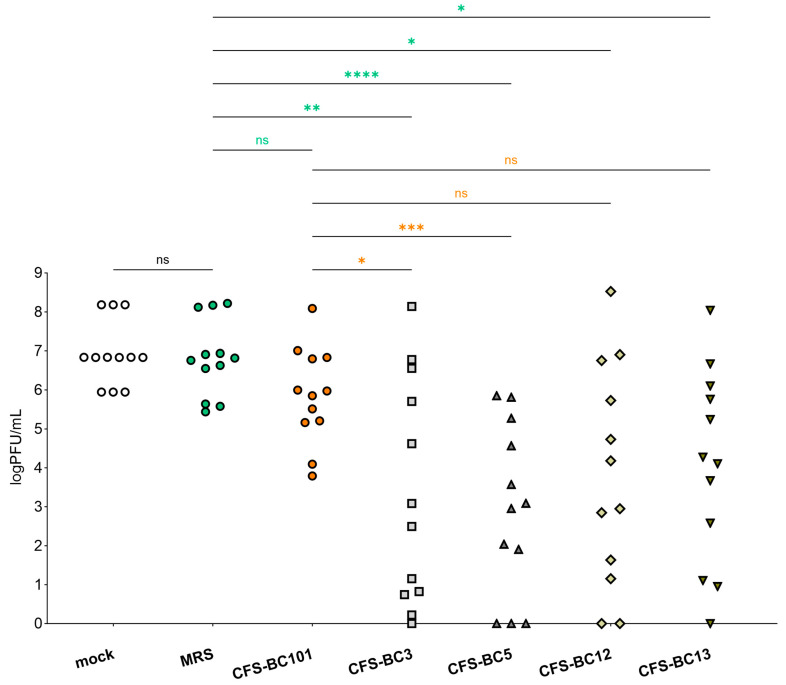
Summary of antiviral activity exerted by CFSs from *L. crispatus* BC3 and BC5, *L. gasseri* BC12 and BC13, *E. faecalis* BC101, and MRS medium at three dilutions (1:2, 1:4, and 1:8) on HeLa and Vero cells. Different shaped symbols were used for the bacterial strains and controls. Significances vs. MRS medium and CFS-BC101 are indicated (* *p* < 0.0332, ** *p* < 0.0021, *** *p* < 0.0002, **** *p* < 0.0001, ns: not significant).

To get a global picture of the inhibitory activity of CFSs on HSV-1 described above, we sum up data obtained in Vero and Hela cells at any time and any dilution (Figure 5). Figure 5 shows a comprehensive representation and the statistical outcome following the comparison of each sample with its own corresponding sample in terms of cell line and time point. The plot shows that there is no statistically significant difference in viral yield between mock samples (untreated virus) and MRS medium-preincubated virus. In addition, CFS from the control strain, *E. faecalis* BC101, had an effect on virus yield that was not significantly different from MRS medium. Oppositely, CFSs recovered from vaginal lactobacilli BC3, BC5, BC12, and BC13 had an inhibitory effect on HSV-1 yield significantly different from MRS medium. In addition, this statistical analysis highlights that only the strains of the *L. crispatus* species (BC3 and BC5) determined an inhibition of viral yield significantly different from that exerted by *E. faecalis* BC101. This last comparison allows the selection of *L. crispatus* BC3 and BC5 as the most promising strains for the antiviral activity profile.

### 2.4. Metabolomic Analysis of Lactobacilli Supernatants

To investigate the reasons for the greater antiviral activity of *L. crispatus* BC3 and BC5 compared to *L. gasseri* BC12 and BC13, we analyzed the metabolic profiles of the CFSs of these lactobacilli, already available thanks to our previous ^1^H-NMR metabolomic study conducted on a larger collection of vaginal lactobacilli and reported in [29]. In the present analysis, we looked for variations in the presence and concentration of metabolites among the four strains belonging to *L. crispatus* and *L. gasseri* species. As expected, the most abundant molecule in all lactobacilli CFSs is lactic acid, which can presumably be considered one of the main causes of viral inhibition. However, there are no relevant changes in lactic acid concentration between *L. crispatus* CFSs (5.10–9.45 mM) and *L. gasseri* CFSs (1.62–9.47 mM) that could support the improved performance of *L. crispatus* strains. Notably, we found that butyrate is produced at most by *L. crispatus* BC5 (0.125 mM), *L. gasseri* BC13 yields only a lower quantity (0.0184 mM), while butyrate is not detected in BC3 and BC12 CFSs. Furthermore, *L. crispatus* BC3 is the only strain that produces 2-hydroxybutyrate (1.525 mM). These data suggest that butyrate and its derivative, hydroxybutyrate, may play a role in enhancing the antiviral activity of strains belonging to the *L. crispatus* species.

## 3. Discussion

Previous reports have demonstrated the ability of *Lactobacillus* species to carry out several beneficial functions in many body areas. Lactobacilli of the female genital tract were shown to exert antiviral activity, e.g., against HIV-1, by means of products they secrete during their growth, either in the bacterial culture medium or in their physiological environment [30,31]. To extend this observation, in the present report we analyzed the antiviral effect of *Lactobacillus* culture supernatants, with a particular focus on HSV-1, a virus that is becoming a prevalent cause of STIs [19,20,24,32]. We designed an experimental setting that recapitulates the physiological situation in the framework of an in vitro study, with its intrinsic limits and approximations. We are aware that the proposed model is simplified as it does not represent the complexity of the vaginal ecosystem in all its components. On the other hand, it allows us to study the interactions between specific physiological components, abstracting them from contextual interferences and providing clear, preliminary information to correctly set up more advanced studies with greater degrees of complexity. In particular, we explored here the specific contribution of individual *Lactobacillus* strains that account for the mutualistic and symbiotic components of the vaginal microbiota. In addition, we took into account that the metabolic products of lactobacilli are diluted in the extracellular milieu following secretion. Thus, we tested CFSs in serial dilutions, and we pre-incubated the virus in the diluted CFSs. This strategy was chosen to be able to ascribe the effect of supernatants to the direct interaction with the virus, excluding effects on the target cells. The possible effects of the complex acidic composition of bacterial cell supernatants on the virus and on the cells were both taken into account. We observed that the supernatants of *Lactobacillus crispatus* are active in inhibiting HSV-1 replication in a dose-dependent manner. They were more effective than other species present in the vaginal microbiota, like *Lactobacillus gasseri*, and in strong contrast with the non-effective supernatants of a pathogenic bacterial strain belonging to *Enterococcus faecalis* species. *E. faecalis* was chosen since its culture supernatant displays a pH value in the range of 3.75–4.12, comparable to those of lactobacilli supernatants. The assay was performed in human HeLa cells, which represent the model of the genital epithelium; interestingly, the same results were observed in simian Vero cells. This is relevant since Vero cells provide a more sensitive readout system, as they can sustain virus replication to a higher order of magnitude. Even if the different patterns in the two cell lines can depend on the complex mechanisms of virus entry into the host cells, which may vary in efficiency or follow diverse pathways (fusion at the plasma membrane or endocytic pathway in a cell line-dependent fashion) [33], overall, the *Lactobacillus* CFSs showed statistically significant anti-HSV-1 activity. Non-specific effects of the supernatants on cells were excluded by carrying out a virus-free assay measuring cell viability after exposure to the bacterial cell supernatants in the same conditions as in the virus-containing experiments. Importantly, no cell toxicity was observed. We can infer that the lactobacilli supernatants affect the virus, although the exact step of the viral infectious cycle that is affected is not known. Further experiments will be needed to elucidate this point.

We carried out a global analysis of the results of virus replication inhibition, to check the statistical strength of our conclusions. The output represented in Figure 5 clearly shows that CFSs of *Lactobacillus crispatus* BC3 and BC5 are the best performers in terms of inhibition of HSV-1 replication. This analysis strongly supports the activity of *L. crispatus* supernatants to hinder HSV-1 replication. In an attempt to highlight which specific metabolites of *L. crispatus* are involved in enhancing antiviral activity, we compared the metabolomes of CFSs of *L. crispatus* BC3 and BC5 with those of *L. gasseri* BC12 and BC13. Interestingly, we noticed that butyrate and its hydroxylated form are peculiar to the metabolism of *L. crispatus* strains. This metabolite, already widely described as crucial for supporting the beneficial activities of probiotics, could therefore also play a role in boosting the antiviral activity. In vivo studies support a protective role of butyrate against viral infections [34]. On the other hand, the picture of in vitro settings is complicated by the different experimental combinations of cell lines and viruses, coupled to the fact that a direct involvement or modulation of the immune system (the major player in vivo) is lacking. Further experiments will help to clarify this point.

## 4. Materials and Methods

### 4.1. Microorganisms and Culture Conditions

The strains *Lactobacillus crispatus* (BC3 and BC5) and *Lactobacillus gasseri* (BC12 and BC13) employed in this study were isolated from vaginal samples of healthy premenopausal Caucasian women, according to the protocol approved by the Ethics Committee of the University of Bologna, Bologna, Italy (52/2014/U/Tess) [29]. *Enterococcus faecalis* BC101 belongs to the Department of Pharmacy and Biotechnology of the University of Bologna. Lactobacilli and *E. faecalis* BC101 were routinely cultured in de Man-Rogosa-Sharpe (MRS) broth (Difco, Detroit, MI, USA) supplemented with 0.05% L-cysteine (Merck, Milan, Italy) at 37 °C in anaerobic jars containing Gas-Pak EZ (Beckton, Dickinson, and Co., Milan, Italy).

### 4.2. Preparation of Cell-Free Supernatants

Overnight cultures of *L. crispatus* BC3 and BC5, *L. gasseri* BC12 and BC13, and *E. faecalis* BC101 were used to inoculate 10 mL of MRS broth at a concentration of 10^6^ CFU/mL. After further 24 h of incubation, the cells were precipitated by centrifugation (10,000× *g* for 10 min) (Centrisart G-16C; Sartorius, Göttingen, Germany), and cell-free supernatants (CFSs) were recovered by filtering the supernatants through 0.22-micrometer-pore-size filters (VWR International, Milan, Italy). CFSs were stored at −20 °C until their use.

### 4.3. Mammalian Cells and Virus

Vero and HeLa cells were grown in Dulbecco’s modified Eagle’s medium (DMEM) supplemented with antibiotics (penicillin 100 IU/mL, streptomycin 100 mg/mL) and 5% fetal calf serum (FCS, Life Technologies, Milan, Italy) at 37 °C in 5% CO_2_.

Herpes simplex virus type 1, strain F (HSV-1 (F)), was previously described [35]. This reference strain was kept frozen at −80 °C in low-passage seeds. Viral stocks were prepared by infecting Vero cell monolayers in T175 flasks with virus seeds at a multiplicity of infection (MOI) of 0.5 PFU/cell and harvesting at 48 h by scraping and pelleting infected cells at 300× *g*. Extracellular virions were collected from the infected cell supernatant by centrifugation at 23,000× *g* for 70 min at 4 °C. Virus titers were determined by plaque assay in Vero cells. For titrations, confluent monolayers of Vero cells, grown in 12-well plates, were infected with serially diluted virus stocks or virions. After 1.5 h of adsorption, the monolayers were overlaid with a medium containing 0.7% methylcellulose (Merck-SIGMA Aldrich, Milan, Italy) and 1% FCS. Plaques were counted after 48 h, and titers were expressed as PFU/mL.

### 4.4. Antiviral Assay

The antiviral activity of *Lactobacillus* supernatants was investigated in Vero and HeLa cell lines. Cells were grown to confluence in 24-well plates (about 2 × 10^5^ Vero cells/well; 6 × 10^5^ HeLa cells/well). CFSs derived from *L. crispatus* BC3, *L. crispatus* BC5, *L. gasseri* BC12, *L. gasseri* BC13, *E. faecalis* BC101, or MRS medium as a control were diluted with DMEM (1:2, 1:4, and 1:8). Viral suspensions were diluted at the equivalent of 0.1 PFU/cell in 5.0 µL DMEM and preincubated for 1 h at 37 °C with 45.0 µL of diluted bacterial supernatants or MRS medium. As a reference (mock pretreatment), the virus was preincubated with 45.0 µL of DMEM alone.

The preincubated virus was then added to 387 µL of DMEM to minimize a direct toxic effect on cells and used to infect two replicate wells for 24 h and 48 h time points. After 90 min of virus adsorption at 37 °C, the viral suspension was removed, and the unpenetrated virus was inactivated by an acidic wash (40 mM citric acid, 10 mM KCl, 135 mM NaCl, pH 3) [36]. Cells were rinsed two more times with PBS, overlaid with DMEM 1% FCS, and frozen at −80 °C at 24 h or 48 h after infection. For virus yield quantification, infected cells were thawed, collected from each well by scraping, and disrupted by ultrasound treatment on ice for 10 s. The progeny virus (intracellular and extracellular) was titrated in Vero cells as above.

### 4.5. Viability Assay on HeLa and Vero Cells

The effect of CFSs from *L. crispatus* BC3, *L. crispatus* BC5, *L. gasseri* BC12, *L. gasseri* BC13, and *E. faecalis* BC101 on the viability of Vero and HeLa cells was investigated by a 3-(45-dimethylthiazol-2-yl)-2,5-diphenyltetrazol (MTT) assay and an erythrosine B exclusion test. For the MTT assay, mammalian cells were cultured as described above and grown to confluence in 96-well plates (Corning Inc., Pisa, Italy). Cells were treated for 90 min (37 °C, 5% CO_2_) with CFSs or MRS medium as a control, diluted in DMEM with 1% FCS to reach the dilution of 1:2 used in the antiviral assay. Afterwards, CFSs/MRS were removed, DMEM 1% FCS was added, and plates were incubated for 24 h or 48 h (37 °C, 5% CO_2_). At the end of incubation, the medium was replaced by 100 μL of MTT (Merck) solution in DMEM 1% FCS (final concentration 0.5 mg/mL), and the plates were kept in incubation for a further 4 h. The formed formazan crystals were dissolved by the addition of isopropanol and quantified by optical density at 570 nm using an EnSpire Multimode Plate Reader (PerkinElmer Inc., Waltham, MA, USA). Results were expressed as percentages with respect to untreated controls. For the erythrosine B exclusion viability test, Vero and HeLa cells were grown to confluence in 24-well plates, treated, and cultured for 24 h and 48 h, respectively, as described above. Cells were then detached with trypsin, stained with erythrosine B solution (0.1% in phosphate buffered saline), and live cells were counted in a Burker chamber.

### 4.6. Data Analysis and Statistical Analysis

All antiviral and cell viability assays were performed in triplicate in independent experiments (*n* = 3), and results were expressed as mean ± standard deviation (SD). To compare viral yield data collected in different experiments carried out with CFSs or MRS medium, the data were scaled to the mean of controls. Scaled data were plotted as mean ± SD.

A two-way ANOVA, followed by Dunnett’s correction for multiple comparisons, was used to analyze the data. To compare antiviral activity data obtained in different conditions (cell line, time point, and dilution), a paired one-way ANOVA was also employed. Statistical analysis was performed using GraphPad Prism version 10.0.2 for Windows (GraphPad Software, San Diego, CA, USA, www.graphpad.com). Differences were deemed significant at *p* < 0.05.

Metabolomic data of vaginal lactobacilli reported in a study by Parolin et al. [29] were queried to search for differences among CFSs from *L. crispatus* (BC3 and BC5) and *L. gasseri* (BC12 and BC13).

## 5. Conclusions

In the present work, we analyzed the inhibitory activity of the supernatants of vaginal lactobacilli against HSV-1 infection. We suggest that the physiological presence of *Lactobacillus* species in the microbiota of healthy women may correlate with an increased ability to control HSV-1 infection or the severity of recurrent disease. Keeping this concept in mind, the more promising *Lactobacillus* strains can be used effectively to reinforce or integrate existing defenses in the female genital tract. In the future, these studies could lead to the design of *Lactobacillus*-based probiotic treatments against the dysbiosis caused by or correlated with STIs. In addition, starting from this work, a variety of other perspectives can be further explored, including the selection of other vaginal *Lactobacillus* strains from healthy women and the assessment of their antiviral activity. This could broaden the arsenal of possible probiotic bacteria with antiviral activity. Second, the prevalence of specific *Lactobacillus* strains could be assessed in the vaginal flora of women who experience genital HSV-recurrent disease. Finally, the in vivo activity of the supernatants could be assayed in mouse models of HSV genital infection.

## Data Availability

Data is contained within the article.
